# Lambda-cyhalothrin ingestion: an infrequent yet concerning presentation of pyrethroid poisoning

**DOI:** 10.1097/MS9.0000000000001246

**Published:** 2023-09-07

**Authors:** Prabhat Silwal, Rojeena Adhikari, Binay Yadav, Sanjan K. Sah, Ajay Bhatt, Samjhana Basnet

**Affiliations:** a Dhulikhel Hospital; b Department of General Practice and Emergency Medicine, Dhulikhel Hospital, Kathmandu University School of Medical Sciences, Dhulikhel, Kavrepalanchowk, Nepal; c Department of Emergency Medicine, John A. Burns School of Medicine, University of Hawaii, Hawaii, USA

**Keywords:** lambda-cyhalothrin, pyrethroid, pesticide, poisoning, case report

## Abstract

**Introduction and importance:**

Lambda-cyhalothrin is a type II pyrethroid compound commonly used as a pesticide, with the potential to cause life-threatening toxicity in humans. Furthermore, among cases of pesticide poisoning in Nepal, organophosphates are most frequently implicated.

**Case presentation:**

A 40-year-old female presented to our hospital after ingesting a pesticide compound with suicidal intent. She also admitted to alcohol intoxication and exhibited symptoms of confusion, abdominal pain, nausea, and vomiting. An atropine challenge test yielded negative results. Therefore, conservative management was continued. It was discovered later that the ingested pesticide was lambda-cyhalothrin. The patient’s condition eventually improved with supportive treatment.

**Clinical discussion:**

Several reports have highlighted the overlapping clinical features between organophosphorus and pyrethroid poisoning. In some cases of pyrethroid poisoning, misdiagnosis as organophosphorus poisoning has occurred, leading to the inappropriate administration of atropine. In our case, initial management was challenging owing to the lack of accurate information about the ingested compound. On further evaluation, cholinergic clinical features were absent and the atropine challenge test was negative. This was suggestive of nonorganophosphorus compound poisoning.

**Conclusion:**

This case illustrates that managing pesticide poisoning becomes challenging when the nature of the pesticide is unknown. Patients suffering from poisoning caused by pyrethroid compounds like lambda-cyhalothrin can present with features resembling organophosphorus poisoning. In such circumstances, a comprehensive clinical evaluation should guide the management. Clinical features and an atropine challenge test can aid in differentiating organophosphorus from nonorganophosphorus compound poisoning. This distinction facilitates therapeutic decision-making, including the consideration of atropine administration.

## Introduction

Highlights

- Lambda-cyhalothrin poisoning is uncommon but can lead to significant toxicity in humans.
- Clinicians face a conundrum when managing cases of pesticide poisoning without accurate information about the involved substance.
- This case demonstrates the importance of a thorough clinical evaluation and an atropine challenge test during the management of such a case.

An estimated 300 000 deaths occur annually as a result of intentional self-poisoning with pesticides in Southeast Asia and the Western Pacific region^1^. Approximately 160 000 people die each year due to unintentional pesticide exposure in the same region^1^.

Although there is no nationwide data on the incidence of pesticide poisoning in Nepal, one study reported the incidence of acute pesticide poisoning to be 62.67 per 100 000 population per year in the Chitwan district of Nepal^2^.

Organophosphorus insecticides are the most commonly involved compounds in pesticide poisoning in Nepal^2–5^. Other compounds involved include aluminum phosphide, zinc phosphide, pyrethroids, carbamates, and organochlorines^2–5^. Lambda-cyhalothrin, a synthetic type II pyrethroid compound, is used as a pesticide and can cause significant toxicity in humans^6^.

Here, we present a case of a 40-year-old female who ingested lambda-cyhalothrin 5% with suicidal intent while under the influence of alcohol. We discuss how the lack of knowledge about the consumed pesticide resulted in challenges during the initial management. We also outline our approach to overcoming these challenges. This case report has been prepared in accordance with the Case Report (CARE) guidelines^7^.

### Case presentation

A 40-year-old female presented to the emergency department 5 h after ingesting ~15 ml of a pesticide compound with suicidal intent. The nature of the pesticide was unknown at the time of presentation. She developed abdominal pain, nausea, and vomiting. She admitted to consuming 500 ml of homemade alcohol 2 h prior to her suicide attempt.

On examination, the patient had a pulse of 120 beats per minute. She appeared confused and had a Glasgow Coma Scale (GCS) of 13/15 with an eye subscore of four, a verbal subscore of four, and a motor subscore of five (E4V4M5). Other findings of the general and systemic examination were unremarkable.

Initially, decontamination was performed by changing the patient’s clothes and washing her body surface. Intravenous fluid resuscitation and continuous monitoring of vital signs were initiated. Given the lack of knowledge about the nature of the pesticide, an atropine challenge test was performed with 1.2 mg of intravenous atropine. The atropine challenge test was negative, as her heart rate increased from 120 to 156 beats per minute. Considering the failed atropine test and absence of cholinergic clinical features, conservative management was continued.

**Figure 1:**
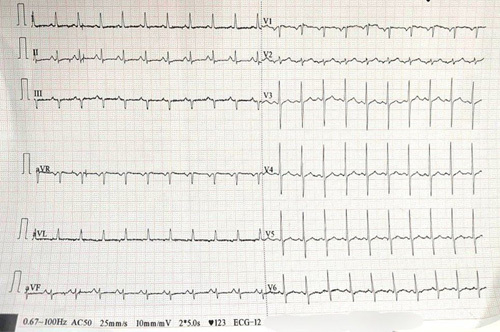
ECG showed sinus tachycardia without any obvious rhythm or wave pattern anomalies.

**Figure 2:**
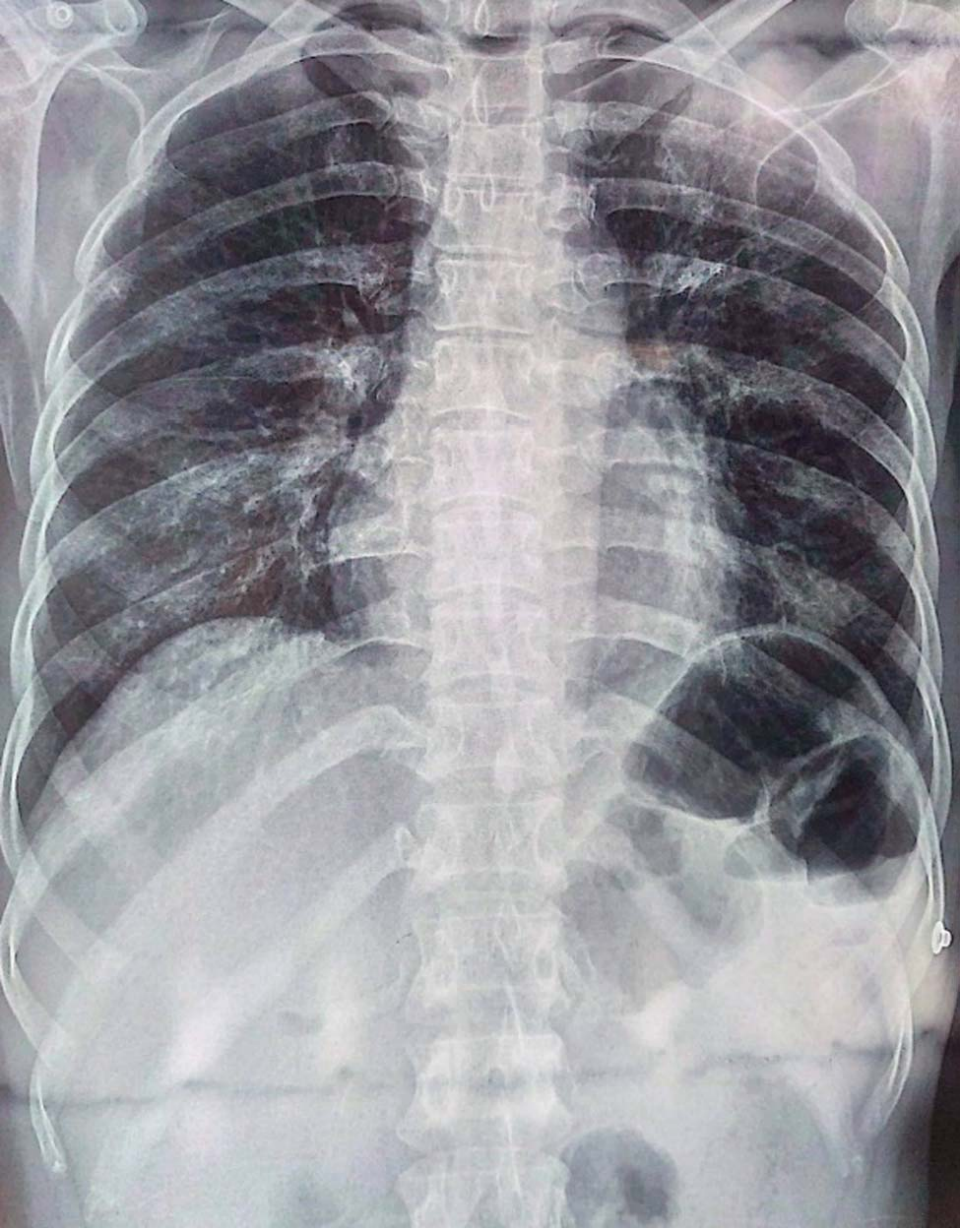
Chest radiograph did not show any distinct abnormal features.

Her laboratory results showed an elevated aspartate transaminase at 120 IU/l (Reference range: <40 IU/l), alanine transaminase 91 IU/l (Reference range: <40 IU/l), and gamma glutamyl transferase 73 IU/l (Reference range: <45 IU/l). ECG showed sinus tachycardia (Fig. 1). Chest radiograph is shown in Figure 2. The patient’s laboratory parameters are detailed in Table 1 and arterial blood gas analysis results are presented in Table 2.

**Table 1:** Laboratory parameters at the time of presentation.

Laboratory parameter	Level at presentation	Laboratory reference range
Total leukocyte count	9300/μl	4000–11 000/μl
Differential leukocyte count:		
Neutrophils	89%	50–70%
Lymphocyte	07%	20–40%
Monocytes	04%	2–8%
Eosinophils	00	2–8%
Basophils	00	0–1%
Hemoglobin	15gm/dl	11–15 gm/dl
Platelet count	240 000/μl	150 000–450 000/μl
Prothrombin time (PT)	12 s	10–13 s
International normalized ratio (INR)	1.0	
Random plasma glucose	80 mg/dl	60–150 mg/dl
Plasma cholinesterase	6546	3930–10 800
CRP quantitative	3.0 mg/l	<10 mg/l
Creatinine	0.5 mg/dl	0.5–1.1 mg/dl
Urea	22 mg/dl	10–45 mg/dl
Serum sodium	138 mEq/l	135–148 mEq/l
Serum potassium	3.6 mEq/l	3.5–5.3 mEq/l
Total bilirubin	0.4 mg/dl	0.3–1.4 mg/dl
Direct bilirubin	0.2 mg/dl	<0.3 mg/dl
Aspartate transaminase (AST)	120 IU/l	<40 IU/l
Alanine transaminase (ALT)	91 IU/l	<40 IU/l
Alkaline phosphatase (ALP)	88 IU/l	42-128 IU/l
Gamma glutamyl transferase (GGT)	73 U/l	<45 U/l

**Table 2:** Arterial blood gas (ABG) analysis parameters at the time of presentation.

Arterial blood gas (ABG) analysis parameter	Level at presentation	Laboratory reference range
pH	7.35	7.35–7.45
Partial pressure of carbon dioxide (pCO_2_)	35.9 mmHg	35–45 mmHg
Partial pressure of Oxygen (pO_2_)	95 mmHg	90–110 mmHg
Bicarbonate	20.1 mmol/l	22–26 mmol/l
Anion gap	18.1 mEq/l	8–12 mEq/l

**Figure 3:**
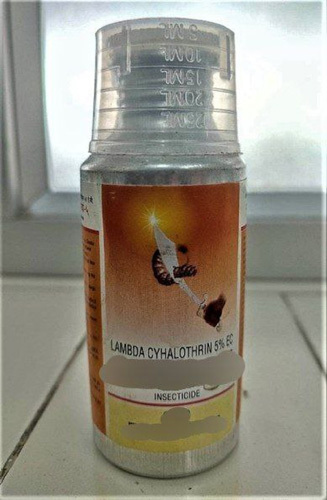
Bottle of the ingested pesticide was labeled lambda-cyhalothrin 5%.

Four hours after presentation, the pesticide ingested by the patient was located and identified to be lambda-cyhalothrin 5% (Fig. 3). Subsequently, the patient was admitted to the medical ward and was managed with intravenous fluids, multivitamin injections, tablet thiamine, tablet pantoprazole, continuous monitoring of vital signs, input/output charting, and a normal diet. The patient’s clinical condition improved, leading to her discharge after 2 days following psychiatric consultation and psychosocial counseling in view of attempted suicide.

A follow-up outpatient department visit occurred 14 days after discharge and a telephone interview with the patient was conducted 1 year after discharge. The patient did not report any medical complaints.

## Discussion

Poisoning caused by pyrethroids is less common than poisoning caused by organophosphorus compounds in Nepal^2–5^. Among pyrethroids, some cases of cypermethrin poisoning are reported from Nepal but a literature search yielded minimal cases of lambda-cyhalothrin poisoning^4^. A study conducted in the United States of America from 2001 to 2005 AD reported that lambda-cyhalothrin poisoning constituted only 3% of the total burden of pyrethroid-related compound poisoning^8^.

Pyrethroid insecticides are categorized into type I and type II based on their chemical structure^6^. Type I pyrethroids are chemical compounds with a basic cyclopropane carboxylic ester structure^6^. The addition of a cyano group results in type II pyrethroids, such as lambda-cyhalothrin^6^. The toxicity of pyrethroid occurs mainly through changes in ionic conduction across voltage-gated sodium channels, leading to a prolonged hyperexcitable state^6–9^.

Clinical manifestations in patients who ingest type II pyrethroid compounds can include nausea, vomiting, tremors, paresthesia, hypersalivation, seizures, and a depressed sensorium^6,9,10^. In our case, our patient suffered from abdominal pain, nausea, vomiting, confusion, and decreased consciousness following the ingestion of lambda-cyhalothrin while under the influence of alcohol. These clinical features could have been caused by the toxicity of the ingested lambda-cyhalothrin, alcohol intoxication, or a combination of both.

The diagnosis relies on the history of ingestion of lambda-cyhalothrin pyrethroid and the presence of consistent clinical features. However, a lack of information about the ingested pesticide can lead to diagnostic dilemmas. This situation arises in a significant proportion of cases of pesticide poisoning in Nepal. A study conducted in Nepal reported that the nature of pesticide was unknown in 14.8% of cases of pesticide poisoning^2^.

Given that organophosphorus poisoning is the most prevalent cause of pesticide poisoning in the region^2–5^ and specific antidotes (atropine and pralidoxime) are available for its treatment^11^, it is imperative that the possibility of organophosphorus poisoning is considered in all cases of unknown pesticide poisoning. However, it is important to note that atropine itself can cause significant adverse effects including blurring of vision, muscular fasciculations, paralytic ileus, pyrexia, tachycardia, arrhythmia, and delirium^11^.

Treatment approaches vary based on the specific compounds responsible for poisoning. Therefore, during the initial emergency management of patients with unknown pesticide poisoning, the differentiation of organophosphorus from nonorganophosphorus compound poisoning is crucial. Difficulties in such differentiation have been previously documented^12–14^. Reasons for such difficulties might include the similar smell of pyrethroids and organophosphates, as well as overlapping clinical features like muscular fasciculations and hypersalivation^14^. A case of pyrethroid poisoning reported from Hong Kong exhibited clinical features resembling organophosphorus poisoning, including bradycardia, salivation, and lacrimation^12^. Another case of pyrethroid poisoning reported from North India was initially treated as organophosphorus poisoning because of a lack of knowledge about the ingested pesticide^13^. A study from China described eight cases of pyrethroid poisoning who developed toxicity following the injection of atropine and one case of pyrethroid poisoning who reportedly died due to atropine intoxication with a total dose of 510 mg, following misdiagnosis as organophosphate poisoning^14^.

Clinical features and an atropine challenge test can aid in distinguishing between organophosphorus and nonorganophosphorus poisoning. Cholinergic features like miosis, bradycardia, increased secretions, urinary incontinence, fecal incontinence, and fasciculations are present in patients with organophosphorus poisoning^11,15^. An atropine challenge test is said to be positive and suggestive of organophosphorus poisoning if the patient’s pulse doesn’t increase by more than 20% or 30 beats per minute following intravenous administration of 1 mg of atropine^11,16^. In our case, cholinergic clinical features were absent and the atropine challenge test was negative. This was suggestive of nonorganophosphorus compound poisoning.

Red blood cell and plasma cholinesterase activities decrease in cases of organophosphorus poisoning and can also be measured to differentiate organophosphorus poisoning and nonorganophosphorus poisoning^6,11,15^. Red blood cell cholinesterase activity may serve as the superior indicator as it has been found to better correlate with the severity of organophosphorus exposure compared to plasma cholinesterase activity^11,15^. However, cholinesterase levels do not have a role in the initial emergency management of organophosphorus poisoning, given the unavailability of timely test results in most cases^11^. Furthermore, these tests may be inaccessible in resource-limited settings. Additionally, it is plausible that alcohol-induced hepatocyte damage and/or lambda-cyhalothrin-associated hepatotoxicity contributed to the raised liver enzyme levels observed in our patient.

No specific antidote for pyrethroid poisoning has been identified thus far. Therefore, treatment should include supportive care, preventing further exposure from the poison source, immediate decontamination by removal of the patient’s clothing and irrigating the patient’s skin with soap and water, administration of activated charcoal (adult dose 50–100 gm) if the patient presents within an hour of ingestion, atropine (0.6–1.2 mg intravenously for adults) if the patient develops hypersalivation, and antiepileptics (diazepam 5–10 mg intravenously for adults) if the patient develops seizures^6,9^. In our case, the patient was managed conservatively with decontamination and intravenous fluids. Multivitamin injections and thiamine tablets were administered in view of possible vitamin deficiencies caused by regular alcohol consumption. Activated charcoal was not administered because the patient presented 5 h after pesticide ingestion.

Life-threatening complications of pyrethroid poisoning can include seizures, coma, and pulmonary edema^6^. Fortunately, these complications did not manifest in our case. Notably, it is reported that simultaneous alcohol ingestion in patients with intentional pesticide ingestion is associated with increased mortality^17^.

A limitation of this case study is the absence of assessments for serum and urine pesticide concentrations. Additionally, tests for red blood cell cholinesterase activity were unavailable in our setting. Furthermore, the dynamic changes in the patient’s serum liver enzyme levels could not be studied, as these tests were not repeated.

## Conclusion

The management of pesticide poisoning becomes challenging when the specific nature of the pesticide is unidentified. In such circumstances, it is crucial to differentiate organophosphorus from nonorganophosphorus compound poisoning. This distinction facilitates therapeutic decision-making, including the consideration of atropine administration.

However, this differentiation can be difficult to achieve. Patients suffering from poisoning caused by pyrethroid compounds like lambda-cyhalothrin can present with features resembling organophosphorus poisoning. In such circumstances, a comprehensive clinical evaluation and an atropine challenge test can aid in ensuring an appropriate and timely diagnosis, as well as proper treatment of pesticide poisoning.

This case demonstrates the need for studies that assess the sensitivity and specificity of cholinergic features and the atropine challenge test in differentiating organophosphorus from nonorganophosphorus poisoning. Moreover, there is a necessity for an updated guideline that comprehensively addresses the management of cases of pesticide poisoning involving unknown compounds.

## Ethical approval

At our institution, case reports require written informed consent from the patient which has been obtained. However, case reports do not require ethical review from the institutional review committee at our institution.

## Consent

Written informed consent was obtained from the patient for publication of this case report and accompanying images. A copy of the written consent is available for review by the Editor-in-Chief of this journal on request.

## Sources of funding

This work did not receive any funding. There are no sources of funding to be declared.

## Author contribution

P.S.: contributed to patient care, conceptualization, design, writing the original draft, reviewing and editing; R.A., B.Y., and S.S.: contributed to contributed to design, writing the original draft, reviewing and editing; A.B.: contributed to conceptualization, writing the original draft, reviewing and editing; S.B.: contributed to patient care, conceptualization, writing the original draft, reviewing and editing.

## Conflicts of interest disclosure

The authors declare that they have no financial or personal conflicts of interest with regard to the content of this publication.

## Research registration unique identifying number (UIN)

1. Name of the registry: not applicable.
2. Unique identifying number or registration ID: not applicable.
3. Hyperlink to your specific registration (must be publicly accessible and will be checked): not applicable.

## Guarantor

Prabhat Silwal.

## Data availability statement

All data that support the findings of this study are included in this article. Further inquiries can be directed to the corresponding author.
